# Embodying togetherness while taking divergent stances. Romantic couples' multimodal positioning practices while performing “we-stories”

**DOI:** 10.3389/fpsyg.2025.1452460

**Published:** 2025-07-11

**Authors:** Stefan Pfänder, Caroline Pfänder

**Affiliations:** Department of Romance Studies, University of Freiburg, Freiburg, Germany

**Keywords:** stance-taking, affective stance, epistemic stance, conversation analysis, embodied practices

## Abstract

Making epistemic and/or affective statements about an interlocutor is a rather delicate endeavor. This is all the more true for spouses who collaboratively tell a good friend a “we-story” about where they met, when they fell in love, how he proposed to her, and that they were not always good partners in everyday life. Using a corpus of 48 collaborative narratives of Italian romantic couples' we-stories, we examine how strong epistemic and affective standpoints interrupt the narrative flow and open up a side sequence in which the delicate positioning of the other is multimodally constructed and negotiated. Using multimodal conversational analysis of three exemplary excerpts, we show how the possibilities of sitting side by side on a sofa while recounting difficult marital episodes affect the interplay of verbal, vocal, and bodily resources in the conversational interaction. Faced with a potentially face-threatening act, participants make use of remarkable multimodal packages to challenge their spouse's unwelcome stance-taking by formulating a counter-stance. These opposing stance-takings then lead to a negotiation and ultimately to a new collaborative narrative that most of the times integrates parts of both (initially divergent) stances. We conclude that a finely nuanced micro-sequential analysis makes it possible to discover the highly complex interplay of multimodal resources like verbal and gestural resonance, mutual nodding, synchronized position shifts, eye contact, choral vocalizations and, maybe most importantly, joint laughter. By reusing, but slightly transforming, these verbal and nonverbal elements from prior talk, romantic partners co-operatively achieve shared epistemic and/or affective stance-taking in collaborative story-telling.

## 1 Introduction

In the embodied practice of jointly telling a friend about where they met, when they fell in love, how he proposed, and about what they quarrel, romantic couples face a severe challenge in talk in interaction. What they are about to tell has been labeled a “we-story” (Gildersleeve et al., 2017; Huber, 2015; Singer and Skerrett, 2014; Strong et al., 2014). We-stories are not easy to tell as they clearly make the stance-taking of both participants relevant, yet, only one person can speak at a time.

One possible solution to this problem that we encounter in our data is that, while one person is telling one of the above-mentioned episodes, their partner employs bodily resources (such as raising their eyebrows, smirking, inhaling deeply, or freezing their upper body, opening their eyes wide etc.) in order to take affective and/or epistemic stances toward the emerging utterance. More often than not, these ephemeral positionings are treated by the current speaker as foreshadowing trouble, or even as conversational challenges. In these cases, the story-telling is momentarily broken off for a side sequence that allows the romantic partners to quickly negotiate how they remember how things took place, and how both of them experienced the narrated event back then, or how they feel about the other person's prior statement in the process of collaborative story-telling.

While taking divergent stances in these side sequences, the partners (in our sample, mostly spouses) not only try to quickly come to a shared understanding of what actually happened, but simultaneously strive at reestablishing both a told and performative stance as a harmonious couple. The maybe most striking result is that, put very simply, while telling you that I don't like what you say, I can bodily show both you and them—i.e., the attentively listening third person as well as imagined later recipients—that we are still in a happy relationship. In a nutshell, then, multimodal stance-taking simultaneously allows for my individual voice to be heard and, at the same time, togetherness to be embodied, such as to make the potentially upcoming anger disappear as fast as possible.

The remainder of this paper is organized as follows: in § 2 we briefly summarize findings on stance-taking in conversation and story-telling. In § 3 we present our data (Italian romantic couples' collaborative story-tellings) and methodology. § 4 explores the semiotic resources used to accomplish an agreement after challenging and negotiating a stance during collaborative story-telling in three exemplary “we-stories”. In § 5 we give an overview of our empirical results and, in conclusion, discuss the implications of our observations for a multimodal conception of stance-taking.

## 2 Stance-taking in conversation and story-telling

Stance-taking is “the public act of positioning oneself toward objects, people or states of affairs” (Andries et al., 2023, p. 1). However, this cannot be done without taking into consideration who we are talking to and what their stance is. Therefore, in his stance triangle, Du Bois (2007) defines the process of stance-taking as “a public act by a social actor, achieved dialogically through communicative means, of simultaneously evaluating objects, positioning subjects (self and others), and aligning with other subjects, with respect to any salient dimension of the sociocultural field” (p. 163). In interactional research, we generally differentiate three types of stance: (1) epistemic stance, (2) affective stance, and (3) deontic stance. Epistemic stance is concerned with our knowledge. Therefore, the questions of primary concern are how knowing we are and how knowing we present ourselves to our co-participants (Couper-Kuhlen and Selting, 2018). However, importantly, our epistemic stance is not fixed, but rather, it is a “thoroughly interactional and emergent process” (Couper-Kuhlen and Selting, 2018, p. 4). Affective or emotional stance, on the other hand, is mainly concerned with how we feel toward an object of stance, our emotions and attitudes toward it (Couper-Kuhlen and Selting, 2018). When being expressed in conversation, these emotions are no longer a personal matter, but they become interactionally relevant through their public display. Lastly, deontic stance is concerned with how desirable an action is.

As has already become evident, stance-taking in interaction is always collaborative, as we usually take into account how our co-participants position themselves toward the stance object (Du Bois, 2007). Therefore, as noted by Kärkkäinen (2006), “stance is very often established and negotiated as an interactional practice” (p. 718; cf. also Bröker and Zima, 2022). The stance we take toward a stance object is thus not fixed, but may change, based on the conditions under which the conversation takes place. For instance, in the case of epistemic stance, it might be important for the participants in conversation to establish who knows what, e.g., if we do not know whether our recipient knows more or less on a specific matter than we do (Satti, 2023a). Interestingly, stance-taking can also be requested by one of the participants, e.g., in a request for verification, where the teller of a story asks his co-teller to verify a specific element of the story delivered to a third party (Hügel, 2012; Satti, 2023b). Through this, tellers can request their co-teller to take a stance on a specific element of the story they are telling, which can either reinforce or challenge the current teller's stance.

Our research will show that when taking a stance in we-stories, co-participants are actually very attentive about what their spouse claims about their shared experiences and attitudes as a couple.

When taking a stance, speakers oftentimes draw on lexico-grammatical resources. Such resources include specific phrases such as “I guess” (Kärkkäinen, 2007) or grammatical markers such as modal verbs (Biber and Finegan, 1989). However, we can express a stance not only by what we say, but also by when we say it. For instance, by chiming into the turn of the person we are talking with, we can express that we share their affective stance, e.g., toward a scenario. Interestingly, in this case, we not only show that we share their affective stance, but we also publicly display that we are equally knowing, which shows that epistemic and affective stances can come hand-in-hand in conversation (Pfänder and Couper-Kuhlen, 2019). However, stance-taking is not a purely verbal accomplishment, but rather, different kinds of multimodal resources can also be used to express one's stance.

What is more, the literature on stance-taking suggests that verbal means of stance- taking rarely make up for a stance act on their own, but rather, they are frequently accompanied by multimodal resources (Andries et al., 2023). Such resources include, for example, prosody (Freeman, 2019), gestures (Yang and Wang, 2025), body movements (Trujillo and Holler, 2021), facial expressions (Ruusuvuori and Peräkylä, 2009), or gaze behavior (Haddington, 2006).

It is, however, interesting to note that verbal and multimodal resources do not always express the same stance, but rather, the body can express a very different stance from what our words do (e.g., Deppermann and Gubina, 2021). This can be done simultaneously, as Andries et al. (2023) have shown convincingly: “The possibility to use multiple semiotic resources (bodily-visual or other) simultaneously, gives rise to a wide range of options for participants to time their stance display, and continuously adapt their stance to that of their interlocutor, without interrupting talk” (Andries et al., 2023, p. 5). In our data of we-stories we more often than not find instances of quasi-simultaneous stance-taking, whereby one of the two only takes a position in terms of body language, which can range from agreement (e.g., nodding, smiling, looking at each other) to astonishment (e.g., putting one's head back, freezing, frowning) to rejection of or dissatisfaction with what is being said.

## 3 Data and methodological procedure

The *Sofa Talks* Corpus (University of Freiburg) comprises 298 video recordings ranging in duration from 10 to 40 min. The corpus data was extracted from as naturalistic a setting as possible, but within an experimental framework. Participants were invited to sit comfortably on a sofa in the presence of a third, well-known friend or relative, and narrate shared experiences. This allowed for a relaxed and familiar environment in which participants could talk comfortably. However, it is acknowledged that collaboratively telling a story in front of a camera, does not replicate everyday life.

Each video contains two participants in a close relationship, be that siblings, friends, or married couples. Both participants were clearly invited to reminisce and tell stories of shared experiences, thus giving both of them equal status in the conversation and equal right to speak. Only if a truly shared experience was discussed, was the video included in the corpus. As a result, the corpus contains 298 video recordings from conversations in German, French, Spanish, Catalan, and Italian. The current study focuses on the Italian data.

In recent years, the expressive bodily resources that contribute to the emergent design of turns and sequences have been taken more and more seriously. This growing body of research is bringing to the open as more and more cases where certain bodily movements recurrently co-occur with verbal expressions in the design of situated courses of action. This systematic interplay of the verbal dimension and embodied elements has been conceptualized as “multimodal packages” (Goodwin, 2007; Pekarek Doehler, 2019; Hofstetter and Keevallik, 2020; Stevanovic, 2021; other authors use the concept of multimodal gestalts; cf. Mondada, 2015; Stukenbrock, 2021). Following Stevanovic (2021, p. 2), we sustain that “an essential feature of such multimodal formations is that none of their single components can achieve the given action on its own. In many cases, these formations become conventional practices for achieving certain goals within a community or activity”.

Some highly conventional practices are recognizable through emblematic gestures,^1^ whereas others (as those under scrutiny here), are more context sensitive, but still recurrent (Satti, 2023a; Ladewig, 2014, 2024).

The crucial importance of the moment-by-moment unfolding of emergent utterance in the real time of interaction has been proven over and over in Conversation Analysis and Interactional Linguistics (Couper-Kuhlen and Selting, 2018). More recently, it has become clear that the bodily expressive movements are equally sensitive to the dialogic temporality (Deppermann and Streeck, 2018). However, the temporality of bodily expressions differs in at least two ways from the temporal design of verbal utterances. First, bodily movements seem to be less bound to turn constructional units. They more often than not start before the verbal utterance and can continue or fade out afterwards. Bodily expression thus can both project or foreshadow verbal expressions that are about to come (Kaukomaa et al., 2014) and they can frame them after the utterance (cf. Pfänder, 2023). Second, the communicative function of bodily movements can be attributed to at least four different dimensions, namely intercorporeality, coordination, common ground, and co-semiosis (for a similar account of interactional dimensions, cf. Pfänder, 2023; and Meyer, 2014). Collaborative story-telling under scrutiny here extensively relies on expressive resources for displaying *intercorporeality*, a shared embodied experience (Tanaka, 2016) of the co-operative actions that unites speakers as they engage (Goodwin, 2018). This requires subtle dynamics of more often than not kinesically achieved (micro-sequential) *coordination*, ensuring that participants know when there is a good moment to take a turn (Deppermann and Schmidt, 2021). Successful interaction can unfold only if *common ground* is constantly being established (Clark and Brennan, 1991; Clark, 1996/2012), meaning that all participants share a similar understanding of what their counterpart is talking about and what they intend to convey. And last, not least, these collaborative story-tellings live by *co-semiosis*, i.e., the collaborative effort to develop the topic of conversation and to create sense together (Schmid, 2020).

For our study, we have made a collection of 48 instances of multimodal stance-taking, of which we discuss—by way of exemplification—three instances in the following Section 4. These excerpts have been chosen to exemplify the three most common types of sequential outcome formats of negotiating stances in our romantic couples' collaborative story-telling sample, namely (a) retracting the counter-stance and agreeing on the initial stance, (b) achieving a new shared stance that integrates parts of both the initial stance and the counter-stance, or (c) slightly changing the topic under discussion.

## 4 Multimodal stance-taking in we-stories

Singer and Skerrett (2014) and Gildersleeve et al. (2017) worked out a concise definition of we-stories: “A We-Story is a type of couple narrative composed by both partners that describes a vivid shared memory. These stories often provide an important image, metaphor, or phrase that serves as a touchstone for the relationship, and they embody the love and commitment each partner feels for the other” (Gildersleeve et al., 2017, p. 314). Consider, for instance, excerpt 1. In the chronological narrative from meeting to marriage, told by the wife on behalf of both of them, she pauses briefly, and then tells how they started to plan their future together, without being engaged, at least formally. Elena utters her epistemic stance: she does not remember that Paolo really proposed marriage. This is where the excerpt^2^ starts:

**Excerpt 1:** ***Marriage proposal***

 ELE = Elena, PAO = Paolo
 **01 ELE: [non] è che c‘è stata proprio una proPOSta-=**
 *      it's not like there's actually been a proposal*
 **02 PAO: [sì.]**
 *      yes*
 **03 ELE: =da +por parte di qualCUn[+o::   ci] spoSIAmo;** **°h**
 *      from anyone               “let's marry”*
    ele:    +pointing gesture @PAO+
 **04 SAR:  **          **[((laughs))     ]**
 **05 PAO: ((smiles broadly @SAR))**

Elena accompanies her statement “it's not like there's actually been a proposal from anyone” [non] è che c‘è stata proprio una proPOSta da (.) por parte di qualCUno with a glance at her husband, who reacts with a questioning look. While eye contact is being made, the third person (SAR), a friend of both, laughs briefly. As a reaction to the laughter Paolo casts a broad smiling glance at SAR. In the meanwhile, Elena then expands her statement and, by animating an imaginary figure, makes it clearer what she meant by her statement, i.e., she gives an account. She has an imagined man (who was not her husband) say quietly: “let's marry” ci spoSIAmo (l.03). This is what in her imagination would have been an “actual marriage proposal”. But instead she remembers that it was “something a bit” … and she searches for the right adjective to complete the sentence (l.06); instead she completes her utterance multimodally, waving her hands and leaving them in the air, as if things were not spelled out clearly, rather the message was somehow “in the air”:

 **06 ELE:  è stato(.) + una cOsa un po[::- °+ ]**
 *       it was kind of like*
    ele:        + palm-up in the air+

In dialogic resonance, i.e., using the same syntactic construction “let's V O”, Paolo animates himself back then and remembers what he said at the time: “Let's buy a house” compriamo CAsa (l.07). His wife confirms this with a smile (l.08).

Paolo continues and evaluates his way of proposing marriage with the assessment: “well that was obvious” be era OVvio (l. 11). To accompany his speech, the speaker performs a “cycle gesture” with both hands (fig. 01), which ends in an “obvious gesture” (cf. Marrese et al., 2021, i.e., “both hands palm up, on hold” as can be appreciated in fig. 02):

 **07 PAO:                 [co ]mpriamo CAsa,**
 *                         “let's buy a house”*
 **08 ELE   ((smiles))**
 **09 PAO:  mhm-**
 *       uhm*
 **10      (1.0)**
 **11 PAO:  be era **%°**OVvi[o. **    **°]**
 *       well it was obvious*
    pao:      %gesture–>
    fig:    °fig.02   °fig.03
 
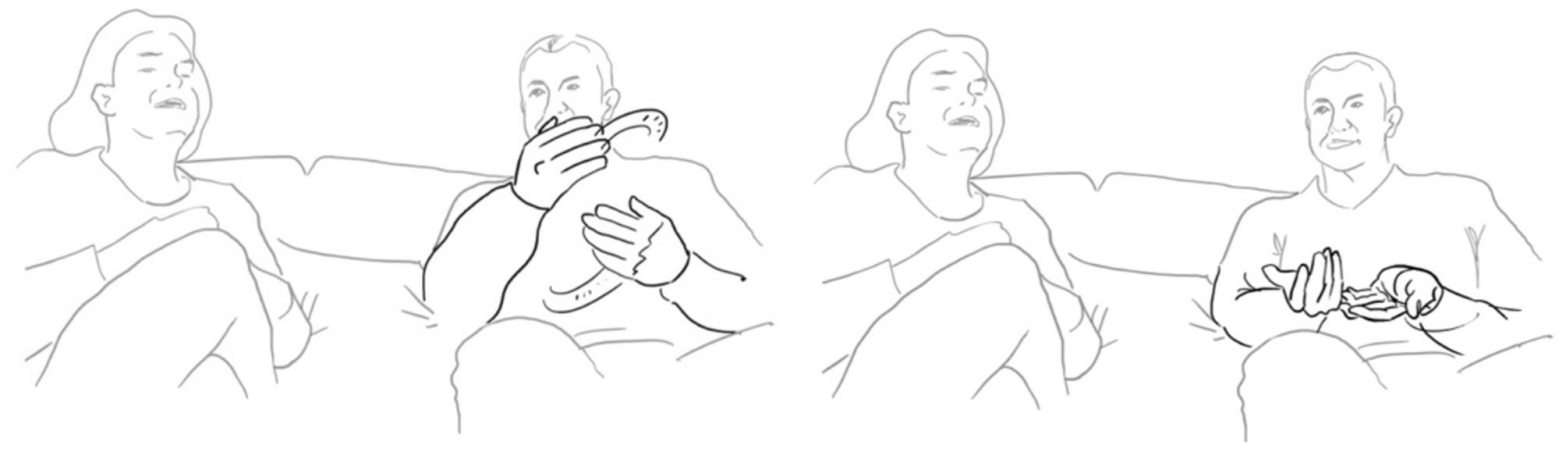

Elena laughs and repeats—each time with an adaptation—both the verbal construction and the gestural dynamic. She utters—now turning back to the third person— (especially in the Italian original) a syntactically very similar but semantically different construction “You could guess it” <<:-)>era da intuIre.> (l. 12). Just like the verbal construction, the gesture begins in a very similar way with a cycle gesture (compare fig. 02 and fig. 04), which, however, does not end in an “it's obvious” stroke as his gesture, but dissolves into a much larger gesture on both sides, and which fits the statement “You could guess it”:

 **12 ELE:   [<<:-)> %e]ra **+**da intuI ° [re.>   ]**
 *       you could guess it*
 *      (lit: it was to be guessed)*
    ele:             +gesture—->
    fed:     —->%
    fig:       °fig.04
 
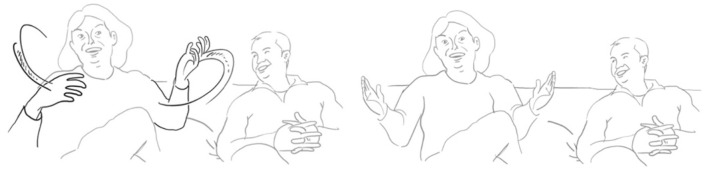
 **13  SAR:       [((laughs))]**
        **[((laughs))]**
 **14  ELE: [((laughs))]**+
     ele:        —->+

A movement of mediation takes place, which works conciliatorily in two directions: first to the partner, then to the camera, i.e., to the public, in the sense of a rehabilitation measure for the partner, which ensures that Paolo does not suffer a loss of reputation in the eyes of the public. Both laugh heartily at this conciliatory moment that ends the side sequence of negotiation with Elena's stance taking in line 15: “It was said between the lines”: era dEtto tra le RIghe.

 **15  era dEtto tra le RIghe;**
 *    it was said between the lines*
 **16  ° hh**
 **17  eh:::-**
 *     umm:*
 **18  <<all> e quindi insomma NIENte;>=**
         *and then well nothing*
 **19 = ci siamo messi a cercAre un po CAse::-**
      *we started looking a bit for houses*

In a nutshell then, the negotiation of stances is done by a variety of instances of verbal and bodily resonance (Brône and Zima, 2014), the opponents repeat and thus reuse the same multimodal resources but change the course of action by coming to a different end and thus expressing a different stance. Since Paolo wanted to buy a house with her, it was clear to him that there was no need for an explicit marriage proposal. Elena, on the other hand, emphasizes that there was no explicit proposal and that her common sense was needed to understand the house purchase as an expression of the desire to enter into a life bond with her, which was only revealed to her between the lines.

Thus, it is only through a multimodal analysis of stance and counter stance (epistemic and affective-evaluative) that we see that this is not a disruptive moment in a couple telling their love story, but rather a humorous form by integrating his cycling gesture with her uncertainty movement in one complex gesture trajectory. As we advocate the view that language is inherently multimodal and thus consider utterances to be of composite nature (Enfield, 2013), verbal and bodily expressive resources are, with Enfield's words, “draw[n] […] together into unified, meaningful packages” (2013, p. 689). The multimodal negotiation of stance-takings ensures that the we-story actually remains a shared story in which the love and commitment for the spouse is expressed. Overall, it can be said that the positioning is found in the verbal wording, the gestures and body movements express the journey from stance to counter stance and, ultimately from separation via negotiation to reconnection.

The next example, then, again shows how couples can agree to disagree, but in a slightly different way. In this example, Valentina and Manolo are talking about how often they met during the time Valentina studied in Milan.

**Excerpt 2:** ***Four times***

VAL = Valentina, Man = Manolo^3^

 **112 MAN:   en tre anni que sono stato gi ù ?**
 *          in three years I came to see you (only*
 *          three times)*
 **113 VAL:  ((laughs))**
 **114 MAN:  %eh NO.**
 *        no way*
       man:   %grappolo gesture—->
 **115 VAL:  ((laughs))**%
       man:           —->%
 **116 MAN:   che bugiarda**
 *        what a liar (you are)*
 **117 VAL:    bon pi ù di TRE o quattro volte non sEi venuto gi ù**
           well you didn't come to see me more than 3 or 4 times
         **[manolo.]**
      ,  manolo
 **118 MAN:  [ma sei ] FUOri;**
         you're out of your mind
 **119 VAL:  ((coughs))**

Having reconstructed that they have been together for 5 years, Valentina and Manolo jointly remember the early years when they used to study at different universities and had to travel several hours by car to see each other.

Valentina then makes the affective, emotional, and somehow deontic-evaluative stance that what had made her insecure about the future of their relationship was that Manolo only came to see her three or four times during the first 2 years. Manolo disconfirms this claim and insists that he came far more often. She then asks him to specify how often he actually came to see her and he gives the unprecise answer of “often enough”. She insists that it had been no more than three or four times, showing this number by tipping her fingers. The use of “only” (solo) has the effect of threatening his face. In order to deal with this injury, he first adopts a dismissive attitude (eh NO), which is then intensified in such a way that he describes his partner as a liar (che bugiarda) and then as someone who is FUOri, i.e., “out of their mind”. Finally, he follows up on her request to know how often he did visit her back then and what he was initially unable to remember (non mi riCORdo), he now can (mi ricordo). She laughs knowingly and challenges him to neatly reconstruct the times he actually came to visit her.

 **120**      **QUANte allora dimmi [tU-]**
         How many times? You tell me then.
 **121 MAN:                 [ eh] non mi riCORdo;**
                     I don't remember
 **122 VAL:  ((coughs))**
** 123     ((laughs))**
** 124 sil:  (—-) **
** 125 MAN:   mi ricordo- [(0.3) ** **   ]**
 *         I remember*
       **VAL:           [((coughs))]**

He takes up her way of illustrating each visit by touching one of the fingers of his hand, giving details about each visit: once it rained, another time there was fog in the street, yet another time was in spring and the fourth time at the end of summer.

 **126 MAN:  allora %una vOlta pioVEva%- =°**
 *        one time it was raining*
       man:    %first finger list%
      fig:   °fig.05
 
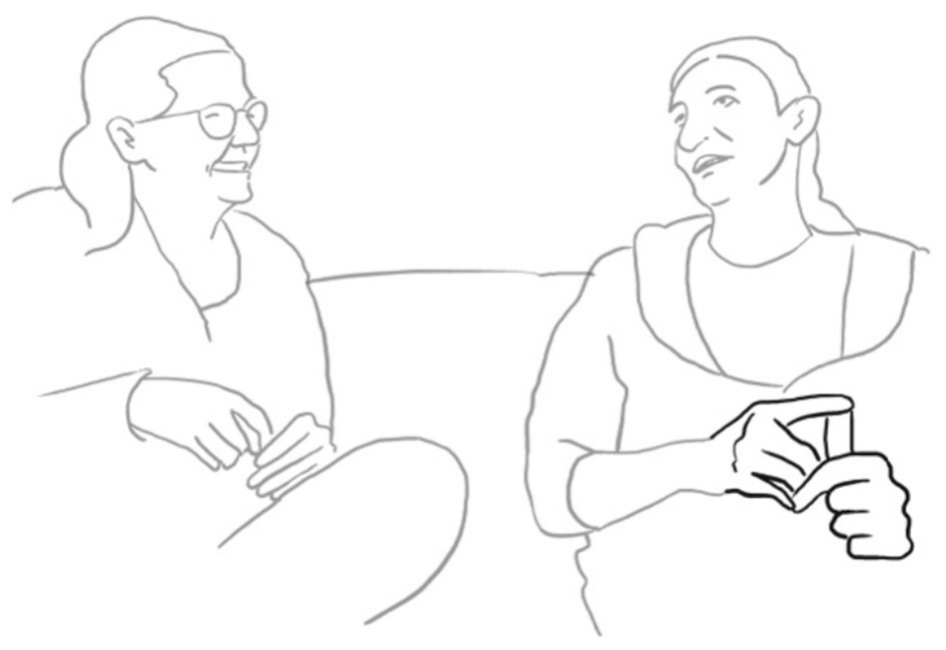
 **127      = %una [vOlta: c'era la NEB <<:-)>bia%°,>**]
           **[((laughs))]**
            *one time it was foggy*
       man:   %second finger list————————————————%
 **128 VAL:   [((risa))                    ]**
       fig:                          °fig.06
 
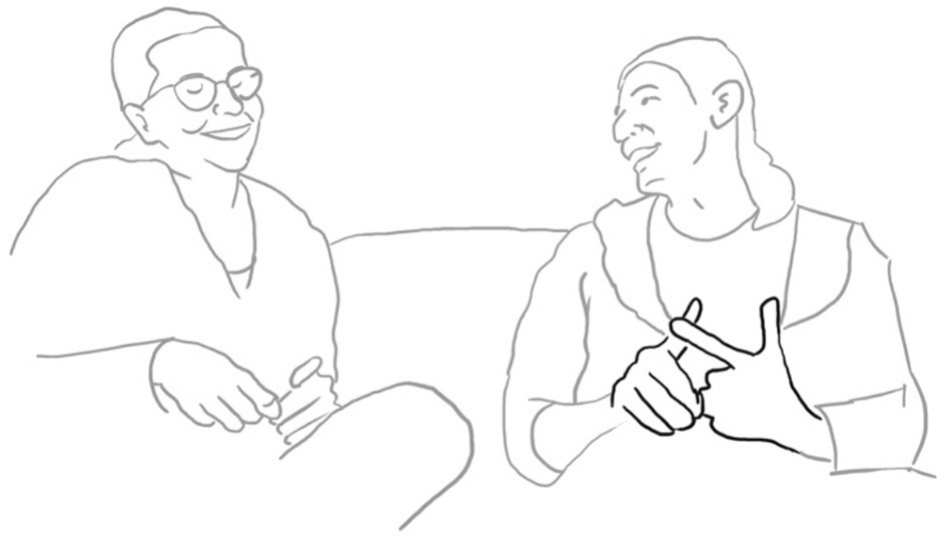
 **129                      [ah bon **  **] SÌ.**
 *                           ah well yeah*
 **130 MAN: %pOi periodi: di primaVEra**%**-°**
 *       then in spring times*
       man:  %third finger list————————%
      fig:                     °fig.07
 
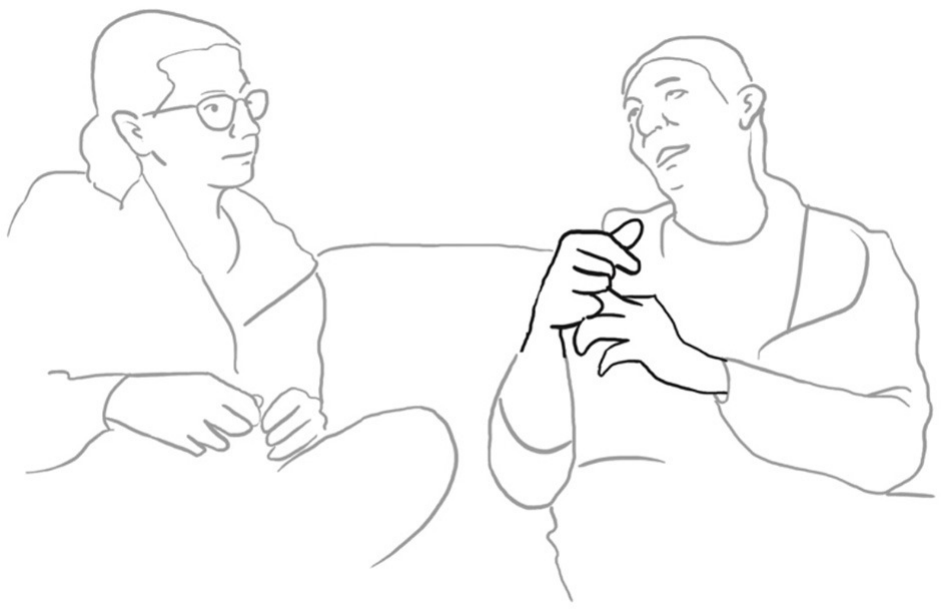
 **131      %e:**  **fine estate   %°**
 *        and at the end of summer*
     man:     %fourth finger list%
     fig:              °fig.08

 
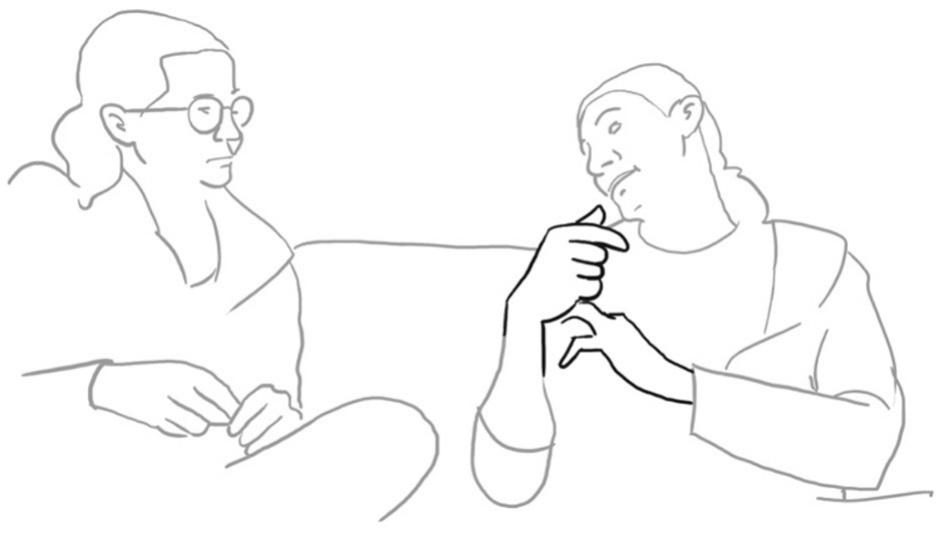
 **132  sil:  (1.6)**

Then she mocks his imitation of her pointing at each finger, counting from one to four and summing it up by a quick gesture covering all her fingers with the other hand, and uttering that this was not really oftentimes.

 **133 VAL:  si ma ° questo una VOLta;(.)**
 *        yes but that (means) one time*
       fig:      °fig.09
 
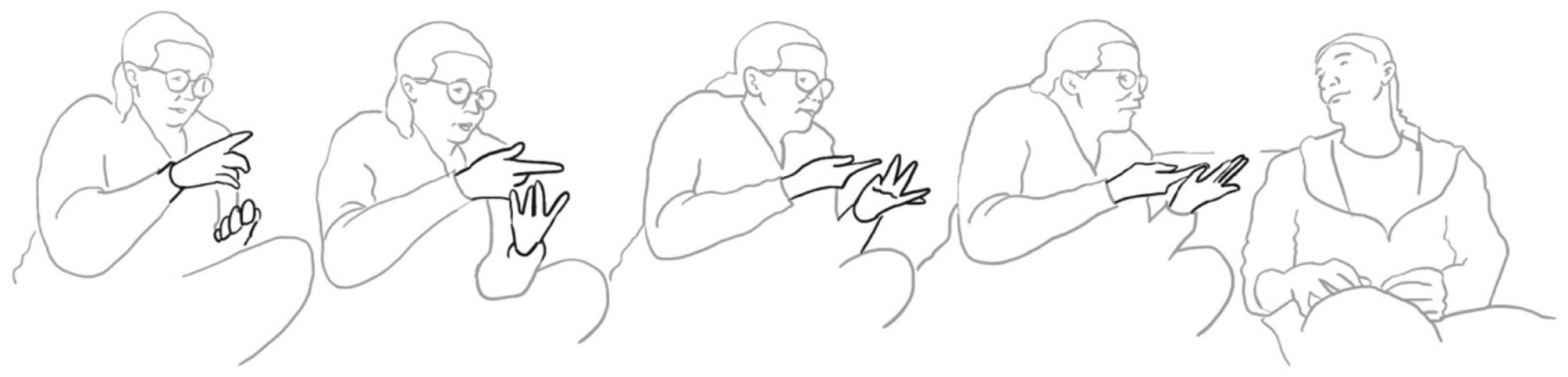

She restarts by saying it was four times and he repairs her statement saying it was six times. They both laugh out loud and start to narratively reconstruct the first week, agreeing that this was a wonderful shared experience. Again, here, we have a long negotiation resolved in an agreement to disagree about how many times he visited her, closing this sequence and opening another sequence of jointly reconstructing the first weekend they spent together. The multimodal character of the negotiation is at first a means of making the epistemic stance literally more concrete, the gesture resonance is then used as a means of mocking via imitation. Thus, carrying out the same gestures leads them to different conclusions which are verbally uttered.

For Valentina this is a complete list of the visits that actually happened; for Manolo, it is only what he remembers, but there were certainly more visits. Posture shows this, he leans back engulfed in the memories while she leans forwards showing him the facts. That is why this sequence ends with 4 vs. 6.

 **134     una per [VOLta non è <<f>che:: > -]**
        *one time per season is not (much)*
 **135 MAN:        [per CUI bon va  ] <<ff>bEh::> adEs[so::- ]**
 *               that's why, well, come on now*
 **136 VAL:                               [quattro]**
        **VOLte.**
 *        four times*
 **137 MAN:  !SEI!.**
 *        six*
 **138 VAL:  comunque.**
 *        whatever*
 **139      ((laughter))**

In a nutshell remember their experience differently which leads them to utter divergent epistemic stances: While Valentina remembers 4 visits, so it was 4 in total, Paolo remembers 4 so there must have been more. She evaluates the number as insufficient. They both count to 4, but for her, it's about the total number, he remembers individual episodes, he remembers the weather, the seasons, etc. By doing so, he protects himself against a potential accusation of misbehavior underlying his wife's stance-taking.

There is one main difference between this example and the previous one about the marriage proposal. Specifically, in this example, we have some movement but very different sense making, while in the previous example, we had movements and utterances, starting out alike but changing on the fly. Then again both examples are very similar in that the multimodality allows the dealing with opposite stances in a humorous way, resulting each time in an agreement to disagree.

In the next example, the situation is slightly different. Here, Angelina and Luigi tell about their time as a newly wedded couple.

**Excerpt 3:** ***She breaks my balls*, …* but only when she is tired***

ANG = Angelina, LUI = Luigi^4^

Angelina begins to tell a story making fun of him just as he made fun of her back then, 2 days after their wedding. Telling this we-story to a third person, her friend, also an Italian woman living in Germany, she starts the stance-taking sequence, polyphonically reenacting his words.

 **39 ANG:  = <<:-)>**   **%mi ha DETto**,**>**°
              *he told me*
   ang:           %puts hand on LUI's shoulder—->
   fig:                       °fig.10
 
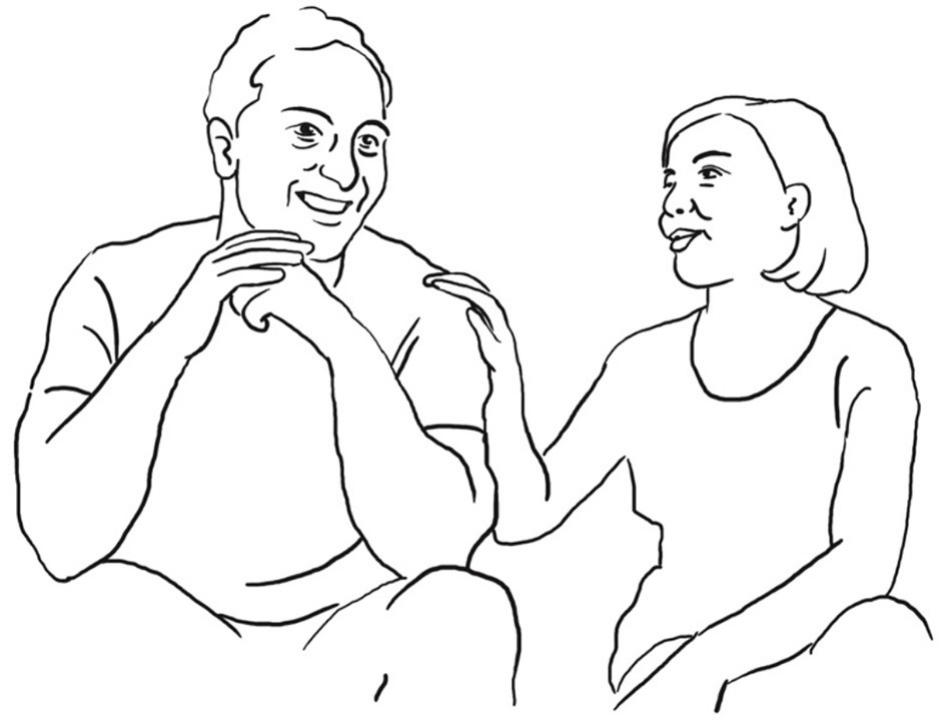
 **40    ° h**
 **41    <<acc> due GIORni dopo che->**
 *          two days after*
 **42    NO:;**
 *       no*
 **43    un po' di PI Ù - =**
 *       a bit early*
 **44    = <<acc> che ci siamo spoSAti->**%
 *          after we got married*
   ang:                  —->%
 **45    sicCOme abbiamo fatto diecimila cose no,**
 *        as we had been doing ten thousand things right*
 **46 SAR: (-)**
 **47 ANG: %e [poi mi] FA,**
 *      and then he says to me*
    ang: %puts hand on LUI's shoulder–>
 **48 LUI:   [ NO: ]**
 *         no*
 **49     che il-**
 *        that the*
 **50 ANG:  SENti-**
 *        Listen*

She literally gets in contact, touching him on the shoulder (fig. 10), doing the good friends gesture, and searching for eye-contact, so that togetherness is established as a solid basis for the now starting, possibly face-threatening reenactment of a conflict encounter only 2 days after their wedding. She explains why she was a bit annoying during the course of the wedding preparations: they had to organize so many things, in her words “ten thousand things” diecimila cose (l. 45), that she possibly got nervous.

He tries several times to establish himself as a co-teller of the sequence and finally manages to take the turn by uttering “no” NO: (l. 48) and at the same time touching her hand on his shoulder (fig. 11).

 **51 LUI: $NO-°**    $
       *No*
   lui:  $touches her hand$
   fig:     °fig.11
 
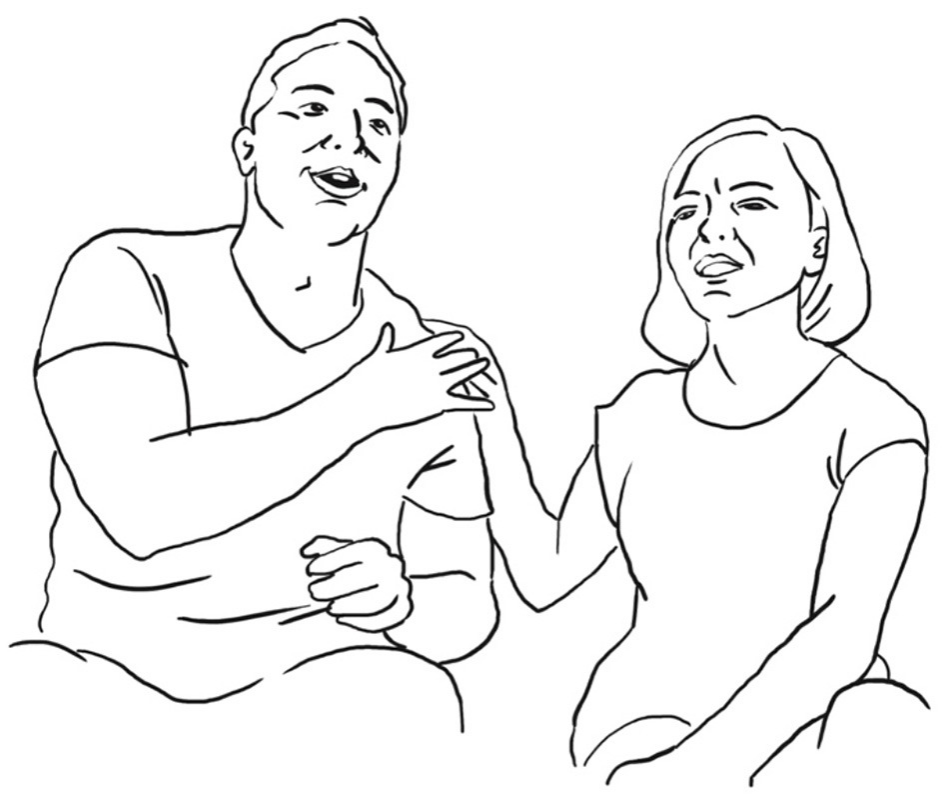
 **52 ANG : <<a> sE il: tuo**% **GRAdo?>**
 *          if your degree*
   ang:          —->%gesture thumb + index finger—->
 **53 LUI:  ° h**
** 54 ANG: di diventAre rompiPALle:-**°
 *      of becoming a pain in the neck*
   fig:               °fig.12
 
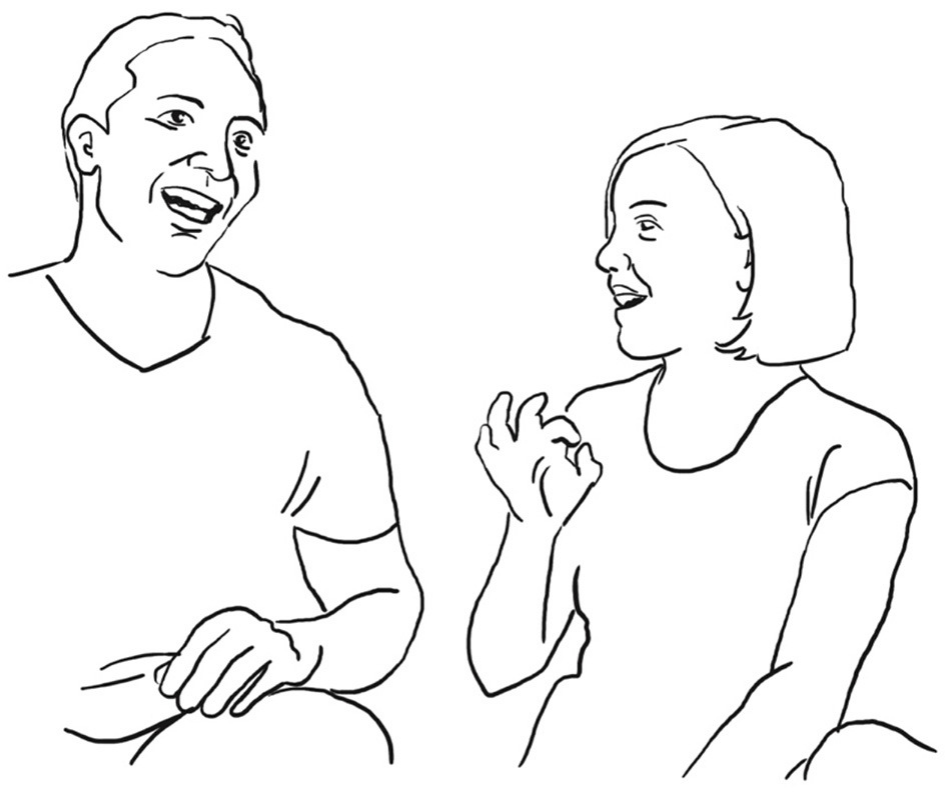
 **55 SAR:  (-)**
 **56 LUI:  <<:-)> he->%**
    ang:       —>%
 **57 ANG:  CREsce- =**
 *         grows*
 **58 LUI:  [in maNIEra esponenziale-]**
 *       in an exponential manner*
 **59 ANG:  [   =così TANto**,**     ]**
             *so much*

Luigi then downgrades her evaluating stance by making an account for her getting annoying, stating that she is only difficult when tired quando è STANca (l. 69). She acknowledges and makes the “precision” gesture (fig. 12). He puts an end to this possibly face-threatening episode uttering NO-, brushing away his wife's gesture (l. 60, fig. 13, cf. Bressem and Müller, 2014):

 **60 LUI:  $[NO-] °**     $
    lui:    $brushes her hand away$
        *no*
    fig:       °fig.13
 
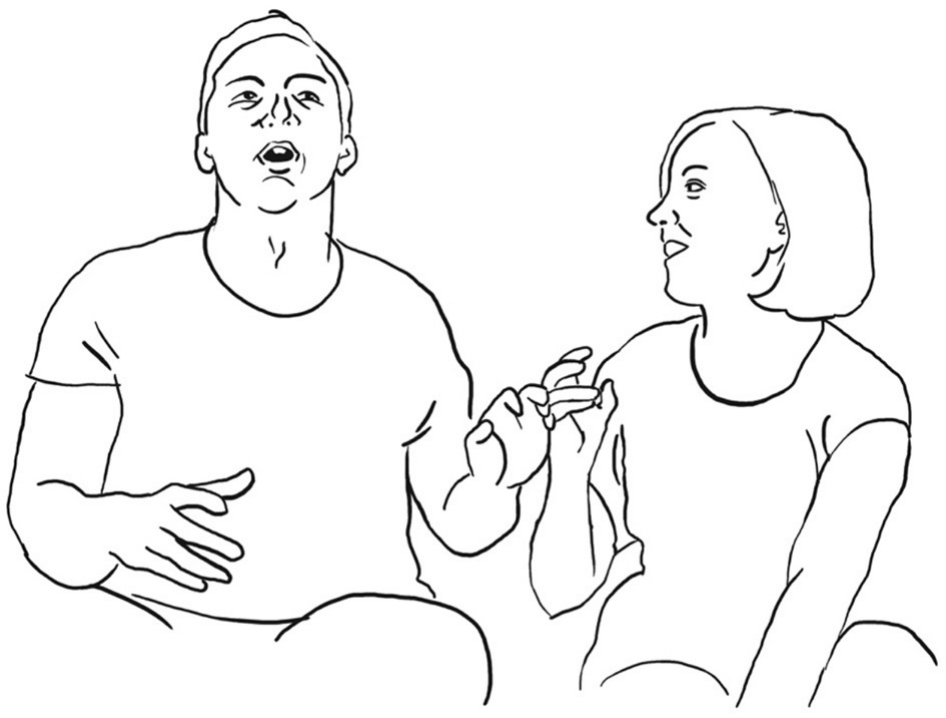
 **61  ANG:  [  es]ponen[ZIAle;** **  ]**
           *Exponentially*

From here, Luigi steps out of the story-telling activity, and goes on describing his partner as una ragazza fanTAStica. This verbal compliment alone is strong enough to express his positive relationship with his wife, no additional touching or physical expression is required, and so he succeeds in overwriting his words that hurt his partner 2 days after the wedding as well as his own injury in the interview (he is presented in a bad light due to his partner's story) with his statement and creating a harmonious atmosphere between the couple, which the public should also experience.

 **62 LUI:       [la veri]t à è che:-**
 *                    the truth is that*
 **63 ANG: ((risa))**
 **64 LUI: ((click))**
 **65      ((click))**
 **66     è una ragazza fanTAStica;**
       *she is a fantastic woman*
 **67 ANG:   oh (.) GRAzi[e; ((risa))]**
       *oh thank you*
 **68 LUI:         [peR Ò :-** ** ]**
        *but*
 **69     quando è STANca-**
       *when she is tired*
 **70 SAR:  (-)**
 **71 ANG:  ROMpo i co (.) eh.**
 *          I'm a pain in the ne(ck)*
 **72 LUI:    [**$**diventa un po' rompi coGLIOni;]**°
        *she becomes a bit of a pain in the neck*
    lui:    $gesture open hands—->
    fig:                     °fig.14
 
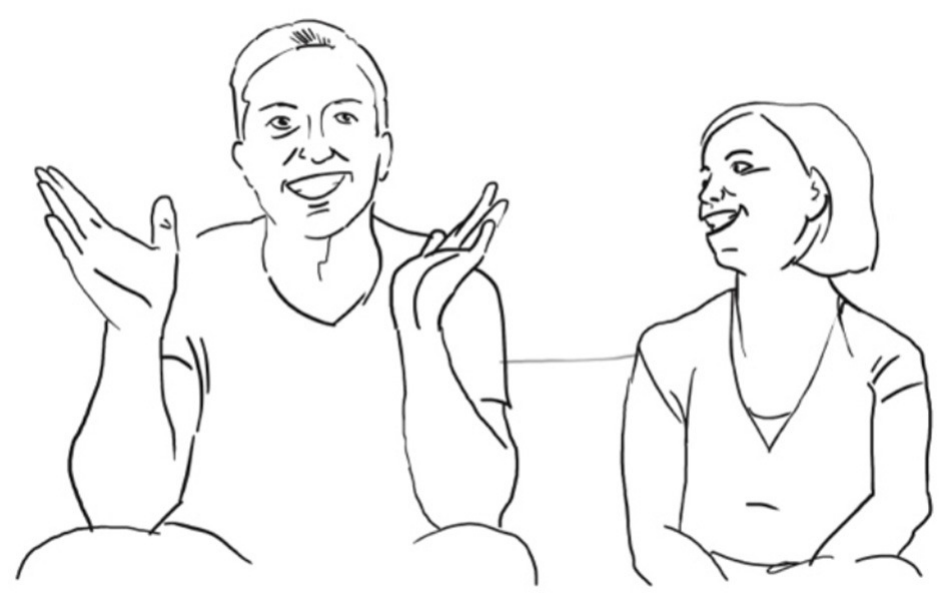
 **73  ANG:   [((laughing))**   **]**

 **74 LUI:  [<<laughing> è STA (.) >]**
 *            and is*
 **75 ANG:    [((laughter))           ]**
 **76 LUI:  [solo quando è STANca; $]**
 *       only when she is tired*
   lui:                 —->$
 **77 ANG: ((laughter)) ° hh**
 **78 LUI: se nO è una raGAZza ° h-**
 *      if not she is someone*
 **79     [una raGAZza: s ì ]:**
 *      someone yes*
 **80 ANG:  [tranQUILla dai;]**
 *      calm right*
 **81 LUI:  [    tran]QUIL[la (s ì ) s-]**
 *           calm yes*
 **82 ANG:           [ ° h      ]**

There is a lot of co-construction taking place throughout the whole micro-sequence. The husband succeeds in taking part in the joint stance-taking action by putting his hand on her hand on his shoulder and uttering “no” several times. Both showing a wide smile. When he has finally won the right to speak, he changes the gaze direction as if to not share the story with his wife, but with his wife's friend sitting opposite them. He avoids gazing at his wife and somehow re-writes their we-story alone.

The plethora of multimodal resources employed by the participants has at least two communicative functions, one is to explicitly establish bodily contact through mutual touch, synchronized wide smiles and eye contact, and second, to negotiate the right to speak and finally to co-construct step by step a shared version of their evaluative stance-taking of her in his eyes, both laughing as he looks into the camera and she in his direction (fig. 14). The observer notes a clear release in the bodily tension as if both participants were happy to have overcome the delicate moment.

As she recounts the story that casts him in a negative light, she touches his shoulder, as if to show: “But I love him anyways”. He touches her back by placing his hand on hers, simultaneously uttering NO:; (l. 42), but does not immediately gain the right to speak. He then completes her emerging sentences, as if to affiliate with her epistemic stance, using this as a means to narrate his own version of the story. Subsequently, she does the same, completing his sentences as if to say: “I, too, know what happened back then”. He overrides her statement about him threatening that, if she continued in that manner… (the actual threat remains unspoken). Naturally, he does not want to be attributed with such a remark 2 days after the wedding, especially on camera. He exits the narrative episode and repositions himself within a general “whenever” structure. She can indeed “break his balls” rompi coGLIOni (l. 72), but only when she is tired. Otherwise, she is a fantastic and calm woman. In this respect, he positions her weakness not as a personality flaw, but as a common human frailty. Overall, the confrontation is characterized by finishing each other's sentences on a verbal level and by gestures of loving connection on a bodily level.

## 5 Discussion

What all our data have in common is that long-married partners talk about themselves as a romantic couple. The flow of narration is interrupted every time one of the two partners chooses a formulation that might be face-threatening to the other. Sometimes, the delicate positioning of the other person pertains to the recounted past, and other times, it relates to the lived present. In all instances, however, it concerns a perceived deficit in the couple's relationship: “He” did not propose marriage (properly), “he” did not visit her frequently enough during their initial infatuation, “he” called her a “pain in the neck” 2 days after the wedding.

In all 48 cases analyzed, the epistemic stance-taking changes as a result of the side-sequence negotiation and the subsequent narrative builds on the slightly actualised story version. The spouses use the negotiation sequence to not only refresh their memory, but also humorously arrive at a story display in which both are in a good position. Moreover, they use this side sequence to publicly demonstrate that they can overcome difficulties in communication, even enjoying it, provoking each other a little and humorously dismissing the provocation.

The individual so positioned does not accept this characterization within the context of the “we-story”. Typically, the discomfort is initially expressed bodily and subsequently articulated verbally. It manifests through more pronounced movements in turning toward and away from each other, in maintaining or breaking eye contact, and, in all cases, it is gestures that ultimately lead to reassurance and thus to the stabilization of the displayed relationship in the here and now of the joint story-telling in front of the camera.

It has been shown that the more positive, joint memory-making experiences a married couple has, the higher the levels of contentment within the marriage (Alea et al., 2015; Alea and Vick, 2010; Gildersleeve et al., 2017). Why do we-stories have such an impact on couples' happiness? Four main communicative purposes to we-stories were suggested by Singer and Skerrett (2014):

- We-stories help to name and structure the routines and values of the couple, thus putting the couple's identity into words.
- We-stories are a way of articulating meaning and purpose for the couple.
- We-stories act as a reminder of the love and commitment between the couple during conflict, thereby allowing for negotiation and growth.
- We-stories are a way of gathering and summarizing the wisdom and experiences of the couple in such a way that it can be transmitted to others.

In our data, we find three possible outcomes of the negotiation: (a) the partner may retract the counter-stance and agree on his wife's initial stance (cf. excerpt “Four times”), (b) the couple may achieve a new shared stance that integrates parts of both her initial stance and his counter-stance (this is what happens in the “Marriage proposal”) or (c) the partner might slightly change the topic under discussion (as in the excerpt “She breaks my balls”). But no matter how the negotiation ends, the collaborative telling is characterized by an affective display of commitment (Tomasello, 2021) and—despite some initial trouble due to divergent stances—finally becomes a moment of embodied pleasure again (cf. Skerrett, 2013, 2016; Skerrett and Fergus, 2015).

Following, Barsalou et al. (2003) and Koch (2013) we understand the concept of “embodiment” as a constituent part of our being-in-the-world: “the body is there from the beginning and movement is what makes it perceptible in the first place” (Koch, 2013, p. 18). In this line of thought, and beyond the classical topic of inferring intention from motion, embodiment can refer to social stimuli (like a possibly face-threatening stance-taking) that cause the activation of bodily resources in counter-stances, but also to the crucial impact of the bodily movements on the sequential progressivity in interaction that are not only perceived by co-participants, but lead to renewed dialogic resonance.

Dialogic resonance (Du Bois, 2014; Zima, 2014) is created by speakers reusing parts of a previous utterance (as shown above in ex. 1 and 2) for activating the perception of similarity and thus connecting utterances that are not necessarily connected on the syntactic level. Building on Du Bois, Warner-Garcia (2013) and, in a similar vein, Chui (2014), transfer the concept of resonance to the analysis of gestures and identify two types of gestural resonance: *collaborative* and *problematising* resonance, both located in different sequential positions and concerning different communicative dimensions such as *co-semiosis* (excerpt “Marriage proposal”), *common ground* (excerpt “Four times”), and *coordination* (excerpt “She breaks my balls…”). In the stance negotiations under scrutiny here, dialogic resonance of a previous composite utterance may occur with an interesting amount of variation at different levels, resulting in varying degrees of similarity and contrast. One of the criteria for resonance is a kind of “active engagement” (Du Bois, 2014) with the previous utterance, as observed in our data. This engagement becomes visible in our analysis through the uptake of parts of an utterance by another speaker to perform their counter-stance (Zima, 2013) in a plethora of *intercorporal* engagements. Note that the narrative flow is interrupted at this point (Satti, 2023a); the partner who has been listening up to this point and who is affected by the face threat makes their own position heard. This opens a side sequence in which both partners ensure that the ascribed action is not concealed at the end, but that it is told in such a way that neither of them looks bad.

Overall, the spouses allow for side-sequences in which stances are negotiated; however, they do not allow for a real quarrel and thus a decisive break in the activity of collaboratively telling their story. Rather, one partner challenges an emergent stance and, subsequently, makes a counter-stance. Embodied resources in a wider sense and embodied resonance in a more specific sense come into play on two observational levels in our study on stance-taking. On the one hand as multimodal packages and on the other in the simultaneity of different statements on the verbal vs. the non-verbal level. In our data, multimodal packages are found as condensations of punchlines in story-telling: the “obvious-gesture” resonance in example 1 for the (un)clear marriage proposal, the resonant “list gestures” in example 2 for his too rare visits, the mutual touch for the challenge (and, ultimately, alteration) of the wife's stance-taking who presented herself as a pain in the neck in example 3. We conclude that the micro-sequential analysis made it possible to discover the highly complex interplay of multimodal resources like verbal and gestural resonance, mutual touch, synchronized position shifts, eye contact, choral vocalizations and, maybe most importantly, joint laughter. These multimodal resources help to “build new action by reusing with transformation [verbal and nonverbal] materials” inherited from prior speakers (Goodwin, 2018, p. 20), and thereby facilitate the romantic partners' co-operative achievement of shared epistemic and/or affective stance-taking in collaborative story-telling.

In a nutshell, investigating an Italian corpus of romantic couples' collaborative story-tellings of how they met, fell in love, proposed marriage, and quarrel as spouses, we found different sequential formats of multimodal packages and gestural resonances that contribute to negotiating epistemic, affective, and sometimes deontic stances. The overall goal seems to be to make a suddenly emergent rivalry friendly again, in order to make one's own individual voice heard, but still accomplish “doing being couple”, i.e., performing the collaborative task as a loving “with” (Mondada, 2024) by “re-writing” the romantic couples' we-stories on the fly.

## Data Availability

The raw data supporting the conclusions of this article will be made available by the authors, without undue reservation.

## Footnotes

^1^Stevanovic (2021, p. 2) gives the following examples: “the striking of the hammer to conclude an auction sale or to initiate a move to a next item in the meeting agenda completing a turn-at-play by placing a token on the game board or formalizing decisions by writing them down” (references omitted).

^2^All three excerpts are transcribed following the GAT2-system, Selting et al. (2009); and Mondada (2018) for the multimodal resources.

^3^Sofa-Talks Ita_2017_Dantoni_02 7.09 – 7.32.

^4^Sofa-Talks Ita_2017_Dantoni_04 chi lava i piatti.
